# Stimulation success!? Improved response inhibition performance after prefrontal single-site and condition-and-perturb transcranial magnetic stimulation

**DOI:** 10.3758/s13415-026-01439-9

**Published:** 2026-04-24

**Authors:** Maximilian A. Friehs, Matteo Ferrante, Hagen Jung, Martin Dechant, Christian Frings, Gesa Hartwigsen

**Affiliations:** 1 Psychology of Conflict Risk and Safety, University of Twente, Enschede, The Netherlands; 2 Max Planck Institute for Human Cognitive and Brain Sciences, Leipzig, Germany; 3 University College Dublin, Dublin, Irelands UK; 4 Institut für Angewandte Informatik (INFAI), Leipzig, Germany; 5 Wilhelm Wundt Institute for Psychology, Leipzig University, Leipzig, Germany; 6 University College London, London, UK; 7 University of Trier, Trier, Germany

**Keywords:** Stop-signal task, Response inhibition, Transcranial magnetic stimulation, Condition and perturb, Prefrontal cortex

## Abstract

In everyday behaviour, the ability to stop an already initiated action is critical for ensuring both your safety and that of others; for example, when stopping a reaching movement towards a hot stove-top after realising it is hot. Neuroscientific evidence points towards the critical role of several regions in the right prefrontal cortex in the coordination and execution of this response inhibition—specifically the right inferior frontal gyrus (rIFG) and the right dorsolateral prefrontal cortex (rDLPFC). The present study investigated the effects of different transcranial magnetic stimulation (TMS) protocols on stop-signal task (SST) performance. We hypothesized that TMS over one or both of these areas would be detrimental to performance. However, contrary to our hypothesis, TMS significantly facilitated performance regardless of the stimulation condition. We applied both frequentist and Bayesian methods to assess the robustness of these effects, revealing consistent reductions in stop-signal reaction time (SSRT) across active conditions. Our results add to the growing body of results that suggest TMS effects may not be as straight-forward as usually assumed and that so-called “inhibitory protocols” can facilitate performance. This result could be explained by a shift in the signal-to-noise ratio depending on the pre-activation of the area. Put differently, TMS may have primed task-related activity in the target areas to a level that was optimal for task performance. Alternatively, the observed effect may reflect an (over)compensation by other parts of the network or disruption of competing resources. Future studies may provide further support for these hypotheses.

## Introduction

Imagine taking your favourite chocolate yoghurt out of the fridge, peeling off the lid—ready to throw it in the bin—and promptly proceed to throw away your tasty yoghurt. Before your dessert reaches its destination and lands in the trash can, you realize that you messed up your actions and are still holding the lid in the other hand. If you have experienced this before, you understand the frustration of being unable to halt an already initiated movement. Conceptually, this scenario exemplifies a broader cognitive challenge: the ability to inhibit an action after recognizing the need to stop. Reactive response inhibition is generally conceptualized as a capacity to suppress, for example, one’s own unwanted actions after recognizing them as currently undesired. This mechanism is key to selecting appropriate responses, suppressing irrelevant stimuli, and achieving relevant goals.

### Response inhibition and the prefrontal cortex

In the laboratory, reactive response inhibition can be behaviourally measured by using the stop-signal task (SST). In this task, participants typically need to make a left-right judgement and indicate the directionality of an arrow presented on a screen; that is, unless a stop-signal is displayed (e.g., coloration of the arrow or a beeping sound). Crucially, the onset of the stop-signal comes after the initial onset of the arrow, thus making it potentially very difficult for the participant to withhold their already initiated action. Several task-specific factors, such as the type of stop-signal, the motivational structure of the game, or the stimuli used, can influence performance (Friehs et al., 2020a, b, 2022, 2024; Kirsten et al., 2023; Markiewicz et al., 2024). With regards to the neural underpinnings of task performance, the prefrontal cortex (PFC) plays a crucial role in response inhibition (Luria, 2012). Key regions in the prefrontal cortex for this process include the right dorsolateral prefrontal cortex (rDLPFC) and the right inferior frontal gyrus (rIFG), which are reciprocally connected and also implicated in other complex cognitive processes (Aron et al., 2014; Callejas et al., 2004; Dambacher et al., 2014; Depue et al., 2016; Miller & Cohen, 2001). Collectively, these facilitate inhibitory control (Aron et al., 2003; Blasi et al., 2006; Verbruggen et al., 2019; Wessel, 2018). Moreover, the right PFC is responsible for modulating activity in the presupplementary motor area (preSMA) and the subthalamic nucleus (Buschman & Kastner, 2015; Chao et al., 2009; Obeso et al., 2013; Rae et al., 2015).

In general, it is assumed that the DLPFC monitors the environment for the need to stop, and once that need arises, it signals the right IFG, which in turn will act as a behavioral “brake” to stop the action (Aron et al., 2004, 2014). Evidence points to the DLPFC being active during and shortly after a task cue (e.g., stopping cue for the present trial) is presented, whereas the IFG is active temporally closer to the actual (stopped) response (Swann et al., 2012, 2013). Furthermore, a recent fMRI study revealed a common neural coding in the right PFC in inhibition tasks across domains (i.e., memory, emotional, and action inhibition) (Depue et al., 2016). More specifically, they showed that the DLPFC is active in all tasks; unlike the IFG, which was only active in tasks requiring response inhibition. Their findings revealed that the IFG receives inputs from the DLPFC whenever stopping is required, and the IFG signals the subthalamic nucleus to stop the motor response.

At the same time, response inhibition is not universally regarded as a unitary, purely reactive process. Several theoretical accounts distinguish between reactive inhibition, which is engaged online following a stop signal, and proactive inhibition, which reflects anticipatory, goal-driven adjustments implemented prior to stimulus onset. Within this perspective, the rDLPFC has often been linked to more proactive or preparatory aspects of control, such as maintaining task goals, monitoring contextual demands, or biasing the balance between going and stopping, rather than directly implementing the stop process itself (Jamadar et al., 2010; Nee et al., 2007). Moreover, alternative frameworks have proposed that inhibitory control may reflect a unitary executive mechanism implemented via attentional processes operating across different temporal scales, rather than separable proactive and reactive systems (Perri, 2020). Within such accounts, prefrontal regions, including the rDLPFC, may influence stopping indirectly by shaping attentional context and task set, thereby constraining the conditions under which reactive inhibition is deployed. Thus, one might argue that the IFG is an area specifically involved in action inhibition, whereas the DLPFC has a broader, domain-general function (Schall et al., 2017).

In summary, based on the current literature, the role of the right DLPFC in the SST could be described as integrating all sensory inputs as well as representing and applying task rules to guide behavior, while the right IFG implements the inhibitory control via its connections to the preSMA and STN (Aron et al., 2014; Schall et al., 2017). Rather than implying a strict hierarchical “chain of command,” this view suggests that the rDLPFC and rIFG contribute to stopping through interacting processes that may operate on partially distinct temporal scales. Therefore, one could argue that this situates the right DLPFC, hierarchically speaking, higher up in the chain of command compared with the IFG. However, these assumptions about the functional relevance of the right DLPFC and IFG as well as their interaction within the prefrontal response inhibition network need to be thoroughly tested.

### Neurostimulation effects on response inhibition

Transcranial magnetic stimulation (TMS) is a noninvasive brain stimulation (NIBS) technique that goes beyond the correlational limitations of neurophysiological recordings and offers the potential to causally validate the roles of key brain regions for specific (cognitive) processes (Bergmann & Hartwigsen, 2020). Previous research has shown that so-called “excitatory” NIBS applied to prefrontal regions can influence inhibitory control alongside other executive functions (for a review, see de Boer et al., 2021; for a discussion of excitatory and inhibitory effects, see Hussain & Freedberg, 2025). Additionally, both “excitatory” and “inhibitory” repetitive TMS (rTMS) protocols have been shown to produce a small but significant improvement in motor impulsivity inhibition, as demonstrated in the Stop-Signal Task (for a review, see Yang et al., 2018; He et al., 2024). Apart from the region over which TMS is applied, the timing as well as the intensity of the stimulation play a key role, although the optimal parameters are often unknown (Hartwigsen & Silvanto, 2023). Optimization can include, for example, a priori electrical field modelling of the induced current flow in the target area to improve targeting and dosing outside the motor cortex (Numssen et al., 2021, 2024; Weise et al., 2022). However, even if all parameters are optimized, it is possible that the intended effect of TMS on performance is not detected. This is, among other factors, owing to potential compensatory effects within a network (Friehs et al., 2021, 2023; Hartwigsen, 2018). For example, in the SST, stimulation over the IFG may not result in the desired performance change, because other areas in the network (e.g., DLPFC, preSMA) may compensate for perturbation and enable maintenance of task performance. A recent systematic review about the manipulation of SST performance with TMS revealed a large heterogeneity within the field about experimental parameters (He et al., 2024). Nevertheless, the most consistent performance impairments were observed for online TMS (stimulation during the task) compared with offline TMS (stimulation before the task), which yielded more null results. These null results may partially be due to compensatory processes after stimulation. Indeed, in a recent study (Friehs et al., 2023), we found that offline perturbation of either the right IFG or DLPFC did not significantly affect performance in a gamified stop-signal task, which we interpreted as compensatory effects within the network. However, the relevance of such potential compensatory effects remains unclear and would require combined perturbation of both areas, which has not been investigated so far.

To address these issues, the present study investigated the functional relevance and interaction of the rIFG and rDLPFC in stopping an initiated action. To this end, we used a "condition-and-perturb" approach, combining online and offline rTMS over both areas. This method involves stimulating one area before task performance and the other during the task, allowing us to assess within-network compensation effects and the interplay between these regions. Crucially, the present study focused on reactive response inhibition by applying online TMS during task performance, thereby targeting neural processes engaged at or immediately following stop-signal presentation. While the rDLPFC has been implicated in proactive or context-setting aspects of inhibitory control, the use of online stimulation in the SST was specifically intended to test its contribution to stopping under conditions that predominantly tax reactive inhibition. We hypothesized that SST performance would decline when either IFG or DLPFC is stimulated, but the network should compensate more effectively for IFG disruption, potentially preventing significant changes in response speed and accuracy. In particular, due to the asymmetry of cognitive organization within the cognitive control network (Bergmann & Hartwigsen, 2020; Friehs et al., 2021; Hartwigsen, 2018), we reasoned that a disruption of either rIFG or rDLPFC could lead to a performance decrease. However, the performance decrease should be larger after rDLPFC stimulation due to the rDLPFC being potentially able to compensate for the rIFG disruption but not vice versa (assuming that the rDLPFC is the more domain general area). Moreover, if both areas are disrupted, compensation should be impaired, leading to stronger performance drop.

## Methods

### Sample

Based on the present study design (i.e., within-subject design with fourfold measurements of SST performance) and our hypothesis, as well as the research on the modulation of cognitive control processes using noninvasive brain stimulation (Friehs et al., 2020a, b, 2023; He et al., 2024), we conducted an a priori power analysis. We assumed an effect size of np^2^ =.1 (f =.33), an α-value of.05 and a power of 1 – β =.95, with a moderate correlation of r =.5 between measurements.^1^ This resulted in a total sample of at least 22 participants. To compensate for potential drop-out, we initially enrolled 40 healthy adult participants (20 females; mean [M] = 30.1, standard deviation [SD] = 5.8; age range: 20–40 years) from the subject database of the Max Planck Institute for Human Cognitive and Brain Sciences in Leipzig, Germany. Participants were examined by a physician to determine eligibility for magnetic resonance imaging (MRI) and transcranial magnetic stimulation (TMS). Exclusion criteria included a history of cardiovascular, neurological, or psychiatric disorders. All participants were right-handed, native German speakers, and had normal or corrected-to-normal vision. They were informed about the experimental procedures but remained blinded to the specific TMS conditions and their sequence. The study received approval from the local ethics committee of the Medical Faculty at Leipzig University and was conducted in accordance with the ethical principles of the Declaration of Helsinki. The study protocol was not preregistered. Written informed consent was obtained from all participants prior to their involvement in the experiment. Of the 40 participants initially recruited, ten were excluded due to significant TMS-related discomfort (n = 4), violations of the stop-signal task (SST) paradigm despite repeated sessions (n = 4), or failure to attend further experimental sessions (n = 2), resulting in a final sample of 30 participants.

### Experimental design

We designed a repeated-measures within-subjects experiment with a multi-site stimulation protocol combining offline and online TMS.

Each session featured a different experimental condition. We included a sham session to establish baseline performances. Moreover, in two separate sessions, participants received stimulation to either the rIFG or the rDLPFC during task performance (online), allowing us to independently assess the effect of focal TMS-induced disruption. Online stimulation consisted of high-frequency rTMS delivered as brief 10 Hz trains (5 pulses) time-locked to the onset of each stop signal. Finally, in a condition-and-perturb session (CP), rIFG was targeted with offline continuous theta burst stimulation (cTBS) before the task, followed by online high-frequency rTMS (10 Hz, 5 pulses) to the rDLPFC during task execution. This condition was designed to probe the compensatory effects of the rDLPFC in response to rIFG disruption, thus probing functional interconnection in inhibitory control processes. Based on the assumed hierarchical structure of the network discussed above, we conditioned the rIFG and perturbed the rDLPFC to optimally test top-down compensation processes. We adopted a condition-and-perturb approach due to the spatial proximity of rIFG and rDLPFC, which made the simultaneous stimulation of both areas impractical. We reasoned that offline cTBS to the rIFG should allow short-term plasticity-driven compensatory mechanisms in the rDLPFC, which were subsequently modulated through online rTMS during task performance.

**Fig. 1:**
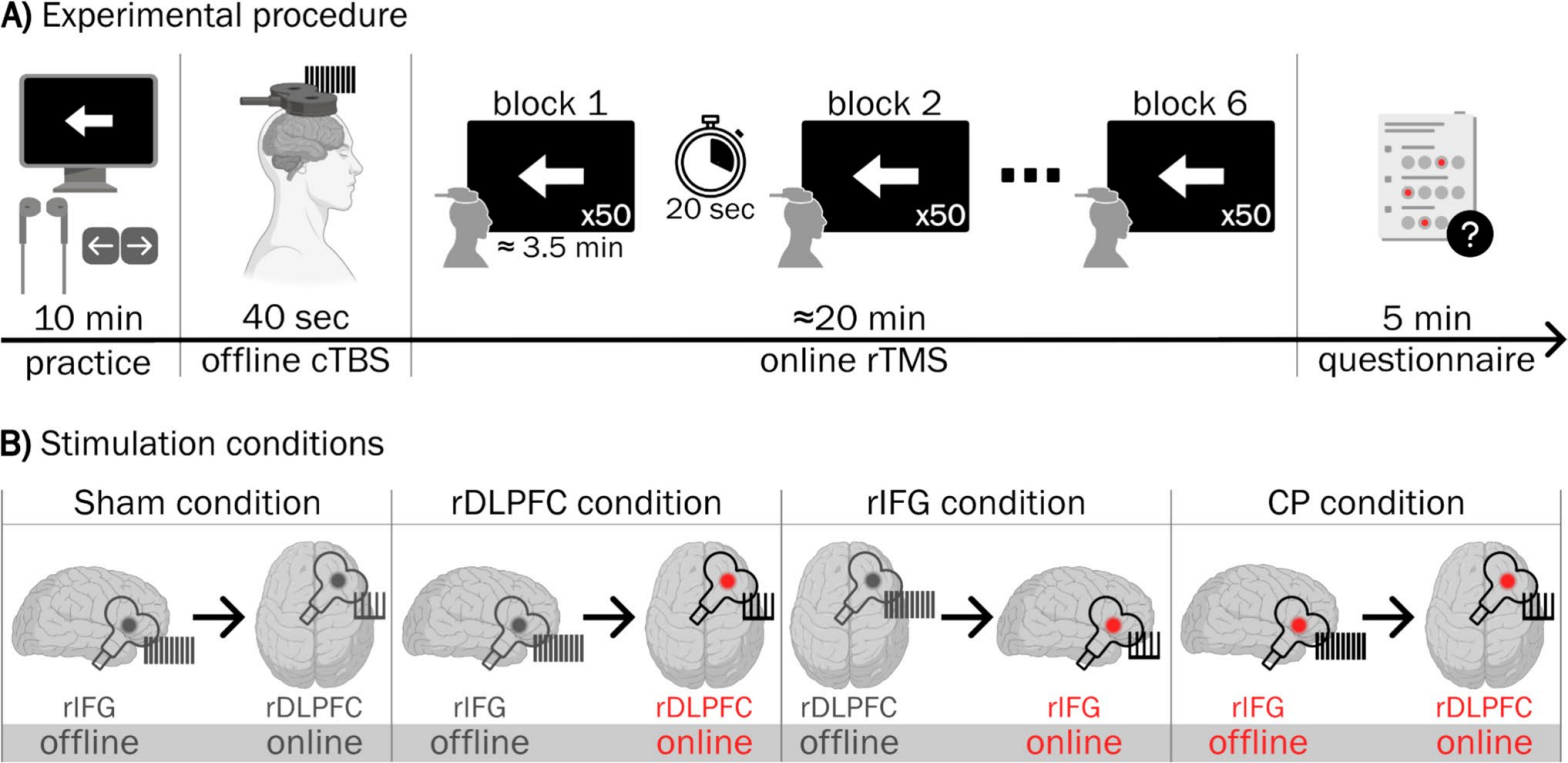
Experimental design. (**A**) Experimental procedure: each session began with a short practice round. Participants received cTBS, immediately followed by the stop-signal task, articulated in six blocks of 50 trials each (38 go, 12 stop; 300 trials total). During the task, online rTMS (5 pulses, 10 Hz frequency) was administered time-locked to the onset of the stop signals. After task completion, participants filled out a self-report questionnaire assessing stimulation-related discomfort and perceived task performance. (**B**) Stimulation conditions: each session was modelled after the condition-and-perturb (CP) procedure. Participants received offline cTBS to one target site and online rTMS (during the task) to the other site. The site targeted for stimulation and whether the stimulation was sham or active varied depending on the condition. In the sham condition, both offline cTBS (to rIFG) and online rTMS (to rDLPFC) were sham. In the single-site conditions (rIFG and rDLPFC), the area of interest was stimulated online, preceded by sham cTBS to the other site. In the CP condition, both sites received active stimulation: cTBS was applied offline to rIFG, and rTMS was delivered online to rDLPFC. Sham stimulation sites are marked in grey, active sites in red. *cTBS* continuous theta burst stimulation; *rTMS* repetitive transcranial magnetic stimulation; *rIFG* right inferior frontal gyrus; *rDLPFC* right dorsolateral prefrontal cortex

To ensure that participants remained blind to the experimental manipulations, each session replicated the structure of CP, incorporating sham stimulation as needed. This was done to preserve the same format across all experimental sessions while varying the stimulation targets, as illustrated in Fig. 1.

The sham condition was kept as the initial session for all participants, while the three active stimulation conditions were counterbalanced across sessions 2–4 following a Latin square pattern with six distinct condition orders (5 participants for each order). Table 1 summarizes the specific combination of stimulation targets for each condition and their respective session order.

**Table 1:** Stimulation conditions and session order

Session order	Stimulation condition	Offline cTBS (before task)	Online rTMS (during task)
1	Sham	rIFG (sham)	rDLPFC (sham)
2 | 3 | 4	rDLPFC	rIFG (sham)	rDLPFC
2 | 3 | 4	rIFG	rDLPFC (sham)	rIFG
2 | 3 | 4	CP	rIFG	rDLPFC

All stimulation conditions were structured like the condition-and-perturb (CP) session to maintain consistency across sessions. The baseline sham condition was always the first session, with active conditions counterbalanced across sessions 2–4

**Fig. 2:**
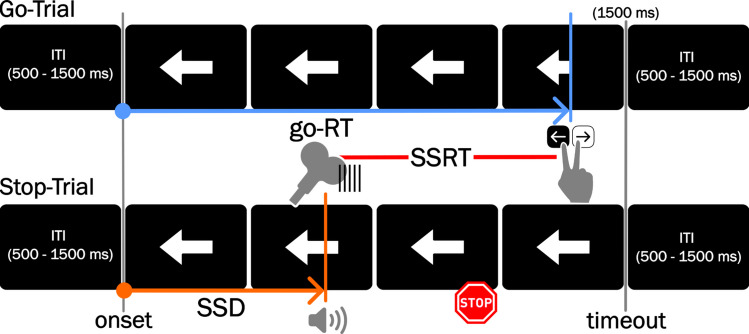
Go- and stop-trials in the Stop Signal Task (SST). Each trial began with a variable inter-trial interval (ITI; 500–1,500 ms), followed by presentation of the go stimulus (left- or right-pointing arrow). The go stimulus remained on screen until the participant responded or until the maximum response window of 1,500 ms elapsed, and it disappeared immediately upon response. During go-trials, participants responded as quickly and accurately as possible by pressing the corresponding key (Go RT). During stop-trials, an auditory stop signal was presented after an adaptively adjusted Stop Signal Delay (SSD), accompanied by simultaneous online rTMS pulses. Participants were instructed to try to inhibit their response when the stop signal occurred, without delaying their responses in anticipation. The stop signal reaction time (SSRT) was estimated using the integration method with replacement of omissions, following Verbruggen et al. (2019). Go- and stop-trials were presented in a 3:1 ratio in randomized order. *rTMS* repetitive transcranial magnetic stimulation

This partial counterbalancing approach was chosen to reduce the number of condition orders while still fitting the repeated-measures design within a sample of 30 participants. Given the limited trainability of the SST task (Hall et al., 2022), we reasoned that keeping one fixed condition would not significantly impact results. We opted to set the sham condition first to establish a clear baseline measurement before any active stimulation was applied. This ensured that the active TMS conditions were not significantly affected by participant drop-out due to low initial performance at baseline. Figure 2 illustrates the sequence of events during go- and stop-trials in the Stop Signal Task (SST) used in the present study.

### Experimental procedure

The experiment comprised four separate sessions, spaced out by a minimum of 7-day intervals to avoid accumulation effects. On the first appointment, prior to the start of the experimental session, participants underwent a resting motor threshold (rMT) assessment, which was used to dose individual stimulation intensity. To reduce the likelihood of mid-experimental drop-out, we also familiarized participants with TMS by applying a small number of test pulses to all stimulation sites. After an introductory training to the SST task, the first session was eventually conducted.

At subsequent appointments, participants completed a practice block prior to the beginning of the session. At the end of each session, participants logged on a questionnaire the stimulation discomfort and impressions on their performance. We used their ratings to manage discomfort and monitor their ability to concentrate and comply with the task requests across the experiment. The first session lasted approximately 3 hr, while subsequent sessions lasted 1.5 hr.

### Task and stimuli

We employed a modified version of the Stop-Signal Task (SST) to assess the effects of brain stimulation on response inhibition. In an SST task, participants respond to a "go" signal, usually a visual cue, while occasionally being asked to inhibit their response when a "stop" signal appears after a short delay. Participants have to balance response speed and accuracy to optimize task performance (Bissett & Logan, 2011). This dual-task design mimics real-world situations where rapid actions can be halted based on the momentary integration of new cognitive input.

In our version of the SST, the go-stimulus was represented by either a right- or left-pointing arrow appearing on a screen, to which participants were instructed to respond by pressing the corresponding key on a keyboard. In stop-trials, an auditory stop-signal followed the presentation of the go-stimulus at a variable delay. Participants were asked to inhibit their response upon hearing the auditory cue. The stop-signal’s timing was controlled by a dynamically adjusted stop-signal delay (SSD), which varied according to the participant’s performance to maintain a 50% inhibition success rate, ensuring that participants operated near their inhibition threshold. Participants were encouraged to respond quickly and accurately to the go-signals, thus avoiding waiting for stop-signals.

Stimuli featured large white arrows displayed on a black background for high contrast, and an auditory stop-signal (a 900 Hz auditory beep lasting 1 s) delivered through noise-isolating earphones. The task was conducted in an acoustically insulated environment, with participants seated at a fixed distance from the screen. The experiment was divided into six blocks (50 trials per block, 300 per session), with 20-s breaks between blocks to minimize fatigue. Each block contained 38 go-trials and 12 stop-trials, randomly distributed. The SSD was initially set to 250 ms and was dynamically adjusted throughout the task.

Performance was measured using a variety of indices. The SSD represents the delay between the go- and stop-signals and was adjusted during task performance by using a staircase procedure. The final SSD used for analysis reflects the value that resulted from this dynamic adjustment. Additionally, we recorded the probability of incorrectly responding when the stop-signal was presented (p(response|signal)), reflecting failures to inhibit a response on stop-trials (stop failures). Other accuracy-related variables, including omission errors (missed responses during go-trials) and choice errors (incorrect responses during go-trials), were also tracked.

Two reaction time (RT)-related measures were recorded: no-signal RT, representing the speed of correct responses on trials without a stop signal; and signal RT, indicating the latency of incorrectly-executed responses on stop-signal trials. We also analyzed the probability of correct inhibition, or the likelihood of successfully halting an initiated action. The main dependent variable was the stop-signal reaction time (SSRT), which was estimated using the integration method with replacement of omissions, as described by Verbruggen et al. (2019), providing a robust reflection of inhibitory control.

### Transcranial magnetic stimulation

Electromyographic (EMG) activity was recorded from the right-hand first dorsal interosseous (FDI), abductor digiti minimi (ADM), and abductor pollicis brevis (APB) using pregelled disposable Ag/AgCl adhesive surface electrodes (15 × 20 mm). Electrodes were placed in a standard belly–tendon montage over each muscle after cleaning the skin with alcohol (Kleim et al., 2007). Signals were amplified (D-360, Digitimer Ltd., UK; 10–2000 Hz band-pass), digitized at 4 kHz (Power1401 MK-II, CED Ltd., UK) and recorded with Signal 4.11. Offline, EMG data were low-pass filtered (sixth-order Butterworth, 500 Hz cutoff), and motor-evoked potentials (MEPs) were quantified as peak-to-peak amplitudes 18–35 ms after the TMS pulse.

The rMT was defined as the lowest stimulation intensity inducing motor evoked potentials of ≥ 50 µV at least five of ten times in the relaxed FDI when single-pulse TMS was applied to the left motor cortex.

Transcranial magnetic stimulation was applied with the aid of stereotactic neuronavigation (Localite, Bonn, Germany). The participant’s head was stabilized with a custom-made silicon mount. Manual navigation was performed to ensure optimal targeting and consistency across sessions: the participant’s scalp was co-registered onto an individual structural MR scan taken from the in-house database or newly acquired. All T1-weighted images were acquired on a 3-Tesla MRI (Prisma or Skyra, Siemens Healthcare, Germany) with magnetization prepared rapid gradient echo (MPRAGE) sequence in sagittal orientation (inversion time = 650 ms, repetition time = 300 ms, flip angle = 10°, field of view = 256 mm × 240 mm, voxel size = 1 mm × 1 mm × 1 mm).

Target locations for rIFG (x = 50, y = 19, z = 16) and rDLPFC (x = 40, y = 32, z = 36) were obtained from previous studies and meta-analyses (Dambacher et al., 2014; Friehs et al., 2023; He et al., 2024; Zhang et al., 2017). These MNI coordinates were converted into each participant’s native space through inversed normalization procedure (SPM 12; Welcome Trust Center for Neuroimaging, University College London, UK).

We used a MagPro X100 stimulator (MagVenture, Farum, Denmark) with an MCF-B65 figure-of-eight coil. A placebo coil (MCF-P-B65) was used for sham stimulation. The coil was oriented at 45° for rIFG and at 315° for rDLPFC stimulation (counterclockwise relative to the mid-sagittal plane).

We employed two TMS protocols. In the CP condition, an offline continuous theta-burst stimulation (cTBS) protocol was applied to the rIFG prior to task engagement to modulate baseline neural activity. Stimulation intensity was set to 90% of each participant’s resting motor threshold (rMT). While this intensity exceeds the classical cTBS protocol of 80% of the active motor threshold (AMT), it is consistent with recent applications of cTBS outside the primary motor cortex and with our previous work using prefrontal theta-burst stimulation (Martin et al., 2023; Williams et al., 2024).

Across participants, rMT corresponded to M = 47% of maximum stimulator output (MSO; SD = 5.45). Offline cTBS was delivered at an absolute intensity of M = 42.23% MSO (SD = 4.63), corresponding to M = 90.22% rMT (SD = 1.13) after rounding to whole MSO units.

In contrast, an online rTMS protocol was applied during task performance to transiently perturb neural activity, with stimulation delivered at 100% rMT. Five pulses at 10 Hz were administered time-locked to the onset of the stop signal (Beynel et al., 2019; Luber & Lisanby, 2014; O’Reardon et al., 2007). To ensure participant comfort, stimulation intensity could be adjusted in 5% decrements, with a maximum reduction of two steps if discomfort or pain occurred. The effective delivered online rTMS intensity across valid active-stimulation sessions was M = 46.8% MSO (SD = 5.27), corresponding to M = 99.93% rMT (SD = 0.57)^2^.

### Data analysis

Our analysis focused on investigating the potential modulatory effects of TMS on the stop-signal reaction time, a well-established index of inhibitory control. Additional behavioral metrics such as go reaction time (Go RT), stop-signal delays (SSD), and various error rates were also evaluated to provide a comprehensive overview of task performance under the different stimulation conditions.

A set of strict inclusion criteria was applied to ensure the quality and validity of the dataset: only participants who completed all four experimental sessions were included; furthermore, we excluded participants whose data violated key assumptions of the independent horse-race model (for more details refer to the supplementary material). Specifically, participants were removed if their mean reaction times on failed stop-trials exceeded their mean go-trial reaction times, because this would undermine the validity of SSRT estimation. We also excluded participants whose probability of responding on stop-trials (p(response|signal)) was either too low (below 0.25) or too high (above 0.75) and those whose go-trial accuracy was insufficient, because poor go performance violates the assumptions of the horse-race model and leads to invalid SSRT estimates (Verbruggen et al., 2019). Finally, participants exhibiting strategic response behaviors, such as consistently delaying go responses in anticipation of stop signals, were identified and excluded from further analysis; proactive slowing was identified through real-time expert monitoring during task performance and confirmed by inspection of block-wise Go RT trajectories, focusing on clear and sustained slowing patterns indicative of waiting strategies. For this criterion, no fixed statistical cutoffs were applied, in line with consensus recommendations (Verbruggen et al., 2019). All participant exclusion took place before any descriptive or inferential statistical analysis was performed.

In line with current best practices outlined by Verbruggen & colleagues (2019), we first computed descriptive statistics for all key performance metrics within each condition. These included SSRT, calculated using the integration method with replacement of omissions; go reaction time (Go RT); mean stop-signal delay (SSD); and three types of error rates: stop failures (on stop-trials), omission errors (missed responses on go-trials), and choice errors (incorrect responses on go-trials). Go-trial accuracy was defined as the proportion of correct responses on go trials (i.e., correct key presses in the absence of a stop signal). Descriptive analyses provided a foundational overview of central tendencies and variability in task performance across the stimulation conditions.

To formally test for differences between stimulation conditions, we conducted repeated measures analyses of variance (ANOVAs) on each of these variables. The main goal of these analyses was to assess whether stimulation conditions had a systematic effect on inhibitory control (as measured by SSRT) and on other performance metrics related to task execution and accuracy. Where necessary, non-parametric tests were employed when data distributions violated the assumptions of parametric testing. Specifically, Friedman tests were used to test for main effects in such cases, and Wilcoxon signed-rank tests were applied for post-hoc comparisons.

As a confirmatory step, we performed Bayesian analyses to quantify the strength of evidence for both the null and alternative hypotheses. Against the background of methodological guidance (Dienes, 2024; Schreiner & Kunde, 2025), we applied both frequentist and Bayesian methods systematically and in parallel, avoiding selective use of Bayes factors. Bayesian inference provides a more balanced approach to evidence accumulation, because it allows support for both the null and alternative hypotheses and avoids overemphasizing trivial effects in large samples (Keysers et al., 2020; Rouder et al., 2009, 2017; Wagenmakers et al., 2018). We used Bayesian pairwise comparisons to assess differences between stimulation conditions in SSRT and other performance measures, computing Bayes factors to compare the relative likelihood of the data under competing hypotheses. Bayesian inference was conducted by using default Cauchy priors (scale = 0.707) via the BayesFactor R package (Rouder et al., 2009).

In addition to these frequentist and Bayesian hypothesis tests, we carried out hierarchical Bayesian modeling within the Dynamic Models of Choice (DMC) framework (Heathcote et al., 2019; Matzke et al., 2013; Weigard et al., 2023). This modeling approach allowed us to estimate latent cognitive parameters underlying task performance, providing a more detailed account of how TMS might influence response inhibition processes at a mechanistic level. The DMC approach was used to model both the go and stop processes and to explore how stimulation affected parameters such as decision boundary separation, drift rate, and nondecision time. The hierarchical structure of the model allowed us to capture both group-level and participant-specific effects of TMS on task performance.

All data processing and statistical analyses were conducted using R. Descriptive statistics, ANOVAs, and nonparametric tests were performed using packages, such as dplyr and afex (Wickham et al., 2025; Singmann et al., 2025), while the Bayesian modeling was implemented using the DMC toolbox.

## Results

**Fig. 3:**
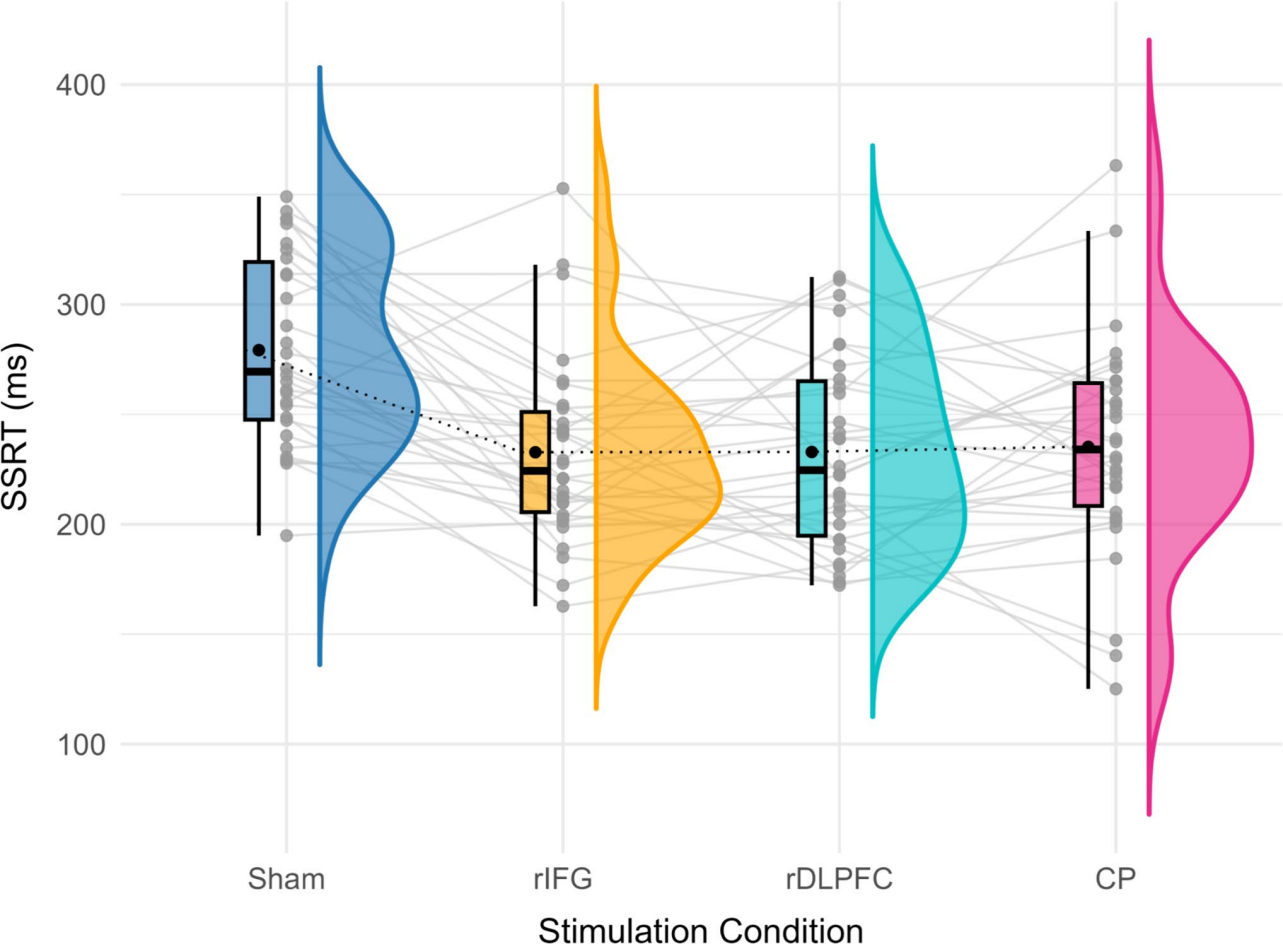
SSRTs across stimulation conditions. SSRTs were estimated using the integration method with replacement of omissions (Verbruggen et al., 2019). Half-violins show data distribution, boxplots indicate the median and interquartile ranges, and grey dots represent individual data. Lines connect individual participant performances, and black dots with dotted lines show group means. *SSRT* stop-signal reaction time

To evaluate the effects of transcranial magnetic stimulation (TMS) on inhibitory control and related task performance metrics, we conducted repeated-measures analyses of variance (ANOVAs) alongside complementary Bayesian analyses. Descriptive statistics for all key metrics across the four stimulation conditions are presented in Table 2. To visualize the effects of stimulation on response inhibition, Fig. 3 shows stop-signal reaction times (SSRTs) across conditions, estimated by using the integration method.

**Table 2:** Behavioural performance metrics across TMS conditions

Metric	Sham		rIFG		rDLPFC		CP	
SSRT [ms]	**279**	±43	**233**	±43	**233**	±44	**235**	±51
Go RT [ms]	**580**	±131	**596**	±163	**633**	±200	**624**	±190
SSD [ms]	**315**	±167	**376**	±166	**412**	±212	**399**	±197
p(response|signal) [%]	**49.0**	±4.74	**48.2**	±3.33	**47.8**	±3.43	**47.7**	±4.80
p(omission errors) [%]	**344**	±0.984	**223**	±0.520	**477**	±0.970	**286**	±0.569
p(choice errors) [%]	**556**	±0.822	**633**	±1.01	**601**	±0.882	**646**	±1.19
Accuracy go-trials [%]	**99.7**	±0.984	**99.8**	±0.520	**99.5**	±0.970	**99.7**	±0.569

Values are presented as mean (in bold) ± standard deviation. Metrics include SSRT, go reaction times (Go RT), stop-signal delays (SSD), error rates, and accuracy measures. Go-trial accuracy reflects the proportion of correct responses on go trials

### Behavioral outcomes

#### Response speed

On average, stop-signal reaction times (SSRTs) were shorter in all active stimulation conditions (rIFG, rDLPFC, CP) compared with Sham, with comparable Go RTs and error rates across conditions (see below for statistical details). Accuracy was consistently high across all conditions, with go-trial accuracy exceeding 99%. Omission errors and failed stop trials were infrequent, indicating reliable and attentive task performance throughout.

Analysis of the stop-signal reaction time (SSRT), estimated using the integration method with replacement of omissions, revealed a significant main effect of stimulation condition, F(3, 87) = 12.60, *p* <.001.^3^ The generalized eta squared (ges) was 0.166, indicating a moderate effect size. Post-hoc pairwise comparisons with Bonferroni correction revealed significantly reduced SSRTs in all active stimulation conditions compared with Sham (rIFG vs. Sham: *p* =.0007, Δ = 46.45 ms; rDLPFC vs. Sham: *p* =.0007, Δ = 46.34 ms; CP vs. Sham: *p* =.0015, Δ = 44.03 ms), indicating enhanced inhibitory control under active TMS. No significant differences were observed among the active stimulation conditions (all *p* = 1.000). Note that when computing an ANOVA across only active stimulation conditions, the F-value was below 0 (specifically: F(2,58) = 0.05, *p* =.951). Bayesian pairwise comparisons supported these findings: Bayes factors confirmed very strong evidence for differences between each active condition and Sham (rIFG vs. Sham: BF₁₀ = 1379; rDLPFC vs. Sham: BF₁₀ = 998; CP vs. Sham: BF₁₀ = 150), and moderate evidence against differences between the active stimulation conditions (rIFG vs. rDLPFC: BF₀₁ = 5.14; rIFG vs. CP: BF₀₁ = 4.96; rDLPFC vs. CP: BF₀₁ = 4.98).

**Fig. 4:**
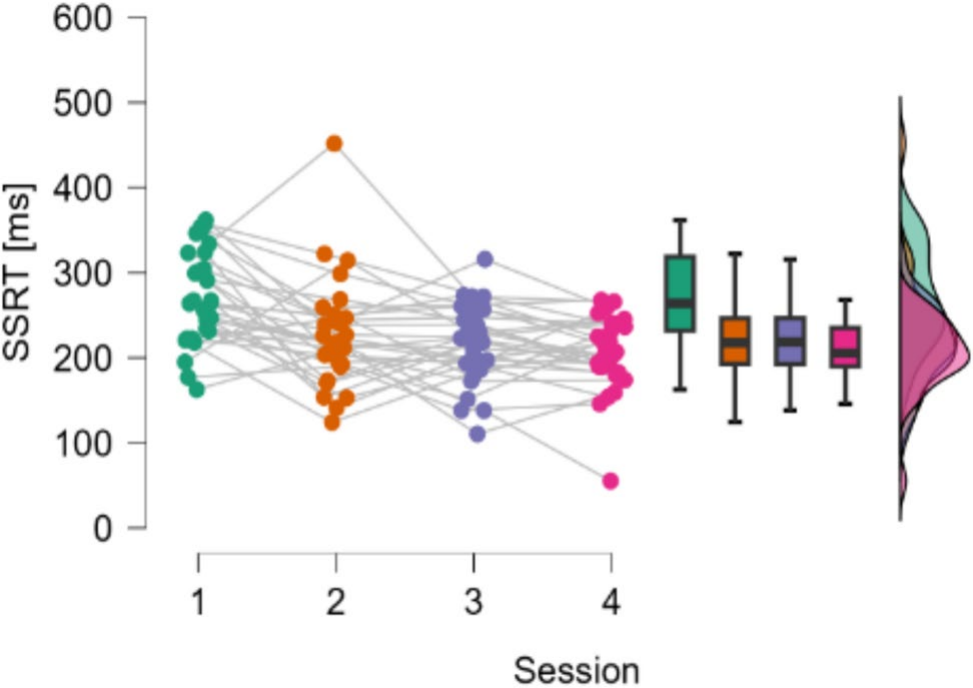
SSRTs as a function of session order. Individual participant SSRTs are shown for each session (1–4), with grey lines connecting repeated measurements within participants. Session 1 corresponds to the sham (baseline) session and sessions 2–4 correspond to the three active stimulation sessions (order counterbalanced). Boxplots indicate the median, interquartile range, and range, excluding outliers; violin plots depict the kernel density of the SSRT distributions. SSRTs are reduced in all active stimulation sessions relative to the sham session, with no further differences among sessions 2–4. *SSRT* stop-signal reaction time

When session order was recoded as a time factor (sessions 1–4) and the analysis re-run, a significant main effect of time was observed, F(3, 87) = 16.15, *p* <.001, ηp^2^ =.36. However, Bonferroni-corrected post-hoc comparisons revealed significant differences only between the first session (i.e., Sham) and each of the subsequent sessions (i.e., active stimulation; all ps <.001), with no significant differences among sessions 2, 3, and 4 (all ps between.38 and 1). This pattern was unchanged when post-hoc tests were evaluated without correction. The corresponding distribution of SSRTs across sessions is shown in Fig. 4.

To further assess variability in SSRTs, we calculated the absolute deviation of each participant’s SSRT from the group mean. Mean deviations were similar across conditions (Sham: M = 36.8 ms, SD = 21; rIFG: M = 32.2 ms, SD = 27.2; rDLPFC: M = 36.9 ms, SD = 22.2; CP: M = 37.3 ms, SD = 33.6). A Friedman test showed no significant differences in deviations across conditions, χ^2^(3) = 2.36, *p* =.501, and Levene’s test confirmed homogeneity of variances, F(3, 116) = 0.25, *p* =.864.

A repeated-measures ANOVA on log-transformed SSD values (corrected to meet assumptions of normality) revealed no significant effect of stimulation condition on stop-signal delays (F(3,112) = 1.07, *p* =.365).

Go reaction times did not differ significantly across conditions. An ANOVA showed no significant main effect of condition on Go RT, F(3, 87) = 1.07, *p* =.365. Descriptively, Go RTs were fastest in the sham condition and slowest in the rDLPFC condition, but these differences did not reach significance. Bayesian pairwise comparisons yielded weak to moderate evidence for the null hypothesis in selected contrasts; comparisons between rDLPFC and CP stimulation (BF₀₁ = 3.8) and between Sham and rIFG stimulation (BF₀₁ = 3.6) suggested moderate evidence for no differences, while the remaining comparisons were inconclusive (BF₀₁ ranging from 0.5 to 1.1).

#### Accuracy

Error rates were consistently low across all conditions. Omission errors (missed responses on go-trials) occurred infrequently, with an average omission rate of less than 0.5%. A nonparametric Friedman test revealed no significant differences in omission errors between conditions, χ^2^(3) = 1.25, *p* =.74. Bayesian pairwise comparisons offered moderate evidence for the null hypothesis, with BF₀₁ values ranging from 4.4 to 4.9. Similarly, stop failures showed no significant differences between conditions, χ^2^(3) = 2.13, *p* =.54. Bayesian comparisons confirmed moderate evidence for the null hypothesis, with BF₀₁ values between 4.5 and 5.1.

#### Hierarchical Bayesian modeling

To complement the traditional behavioral analyses, we modeled latent cognitive processes using a hierarchical Bayesian race model implemented within the Dynamic Models of Choice (DMC) framework (Matzke et al., 2013). Models were estimated using the standard prior specifications provided in the DMC toolbox (Heathcote et al., 2019), without modifying or introducing any custom priors. This approach provided estimates of trigger failure probability (P(TF)) and go failure probability (P(GF)). Trigger failure reflects the likelihood of failing to initiate the stop process on stop-trials. In contrast to stop failures, which occur when the stop process is initiated but fails to win the race against the go process, trigger failures indicate that the stop process was never triggered at all. Go failure (P(GF)) represents the probability of failing to initiate the go process on go-trials. While similar in outcome to omission errors (missed responses), go failures reflect latent cognitive failures to trigger a response, rather than merely observable behavioral lapses.

Analysis of P(TF) revealed low failure rates across all conditions. Although density plots suggested slightly higher P(TF) values in the sham condition and lower values in the CP stimulation condition, these differences were not statistically significant. A repeated-measures ANOVA found no main effect of stimulation condition on P(TF), F(3, 87) = 1.42, *p* =.24. Bayesian comparisons further supported this conclusion, with Bayes factors providing weak to moderate evidence in favor of the null (rIFG vs. Sham: BF₀₁ ≈ 0.72; rDLPFC vs. Sham: BF₀₁ ≈ 1.64; CP vs. Sham: BF₀₁ ≈ 2.58).

Similarly, go failure probabilities (P(GF)) remained low and stable across all conditions. A repeated-measures ANOVA yielded no significant main effect of stimulation condition, F(3, 87) = 0.89, *p* =.45. Bayesian comparisons again favored the null hypothesis, with Bayes factors indicating moderate support for no differences (rIFG vs. Sham: BF₀₁ ≈ 3.98; rDLPFC vs. Sham: BF₀₁ ≈ 1.35; CP vs. Sham: BF₀₁ ≈ 2.92).

**Fig. 5:**
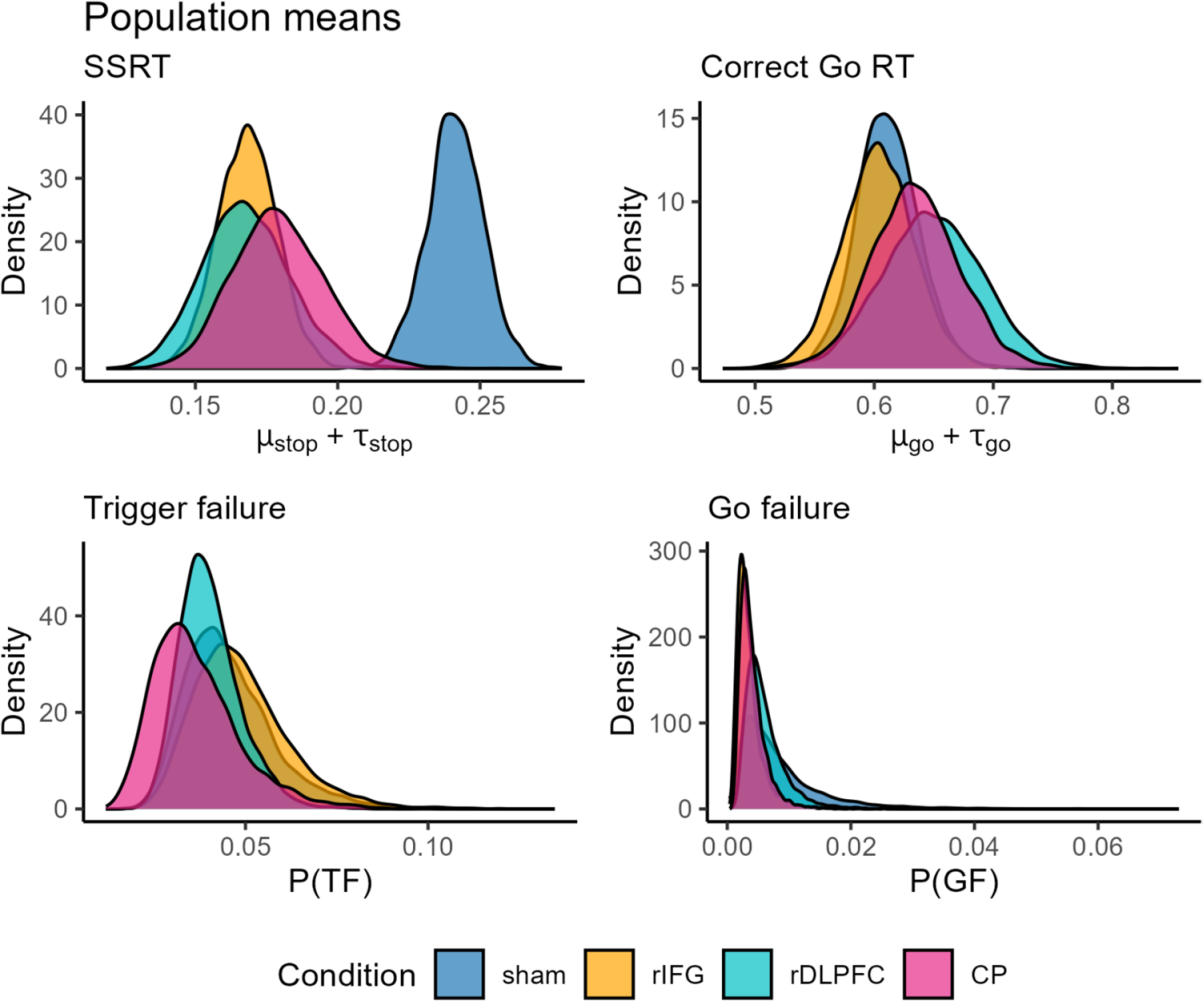
Posterior distributions of SSRT, Go RT, trigger failure (P(TF)), and go failure (P(GF)) across stimulation conditions. Active stimulation resulted in reduced SSRT and trigger failure rates compared with sham. *SSRT* stop-signal reaction time; *RT* reaction time

The hierarchical Bayesian model closely fitted the observed data across conditions. Posterior distributions confirmed that SSRTs were reduced under all active stimulation conditions compared to Sham, with no meaningful differences between active stimulation conditions (posterior probabilities for pairwise comparisons ≈ 0.3–0.5). Bayesian pairwise comparisons indicated high probabilities for improved inhibitory control under active stimulation relative to Sham (posterior probability *p* > 0.99). Figure 5 depicts the posterior distributions for SSRT, Go RT, P(TF), and P(GF) across stimulation conditions.

## Discussion

In the present study, we investigated how TMS over the right IFG and DLPFC, as well as combined CP stimulation, influences response inhibition and network adaptation. We expected a staggered decrease in performance depending on the TMS condition. However, contrary to our expectations, all active TMS conditions led to a performance improvement, compared with sham stimulation. Moreover, there was no difference between the active TMS conditions. Consequently, the present results are an exception to the findings that online rTMS has a disruptive or no effect on performance. Given that the present study shows a counterintuitive improvement of performance, future research into the exact dynamics of TMS effects on reactive response inhibition is warranted. Hierarchical Bayesian modeling of latent cognitive processes supported these findings. Bayesian modeling revealed no differences between active stimulation conditions in stop- or go-trigger failure probability. Specifically, trigger failure (P(TF)) and go failure (P(GF)) probabilities were low and stable across conditions, suggesting that enhancements in stopping performance were not due to strategic shifts, lapses in process engagement, or superficial behavioral artifacts. This suggests that the facilitation of SSRT under stimulation is unlikely to be explained by differences in the probability of launching the stop and go processes. This result contrasts with our initial hypotheses: we expected a reduction of performance in response to TMS, with the largest performance decrease after condition-and-perturb TMS to both areas. These findings add to the growing body of literature that shows heterogeneous TMS effects. In fact, a recent review found that a large number of TMS applications that should impact response inhibition lead to no performance difference and especially the results pattern for offline TMS is inconsistent at best (He et al., 2024). Specifically, offline TMS (either cTBS or 1 Hz TMS) leads, more often than not, to no effect on performance at all. The effects for online TMS (e.g., single-pulse or 10-Hz protocols) seem to be more consistent in disrupting performance, although there is variation as well. When it comes to consistency, in our study, the SSRT-variance in the CP-condition was descriptively larger compared to the other conditions. Although the difference was not statistically significant, it may indicate that the potential for performance modulation (and therefore TMS-specific impact on the network) is largest in the CP TMS condition.

### Implications of our findings

Several factors may explain the unexpected performance facilitation following different TMS conditions compared with baseline in our study. Transcranial magnetic stimulation is often assumed to disrupt processing in a specific area when applied over the prefrontal cortex, but it does not always seem to be this clear-cut (Hussain & Freedberg, 2025). For example, a protocol that is assumed to be disruptive in the cognitive domain when stimulating the PFC could be expected to be facilitatory in the motor domain.

In fact, TMS effects in general may not be easily grouped into “inhibitory” and “facilitatory” protocols, especially when considering TMS effects on different brain areas and cognitive processes (Walsh & Cowey, 2000). The measurement of TMS effectiveness when considering changes in corticospinal excitability via motor-evoked potentials (MEPs) as the output measure is straight-forward, and an increase or decrease of MEPs can be directly linked to the stimulation protocol. In contrast, for cognitive processes, a direct outcome measure is lacking and the assumed chain of causality between the stimulation protocol and the outcome measure is influenced by numerous intermediate factors and processes (Bergmann & Hartwigsen, 2020). Moreover, cognitive processes are distributed across multinode networks and the role of different nodes for different subprocesses is often unknown. Another factor that complicates the predictability of TMS effects, especially for cognitive functions, is the relevance of the current brain state for the outcome, which is commonly referred to as state dependency (Silvanto et al., 2008; Silvanto & Cattaneo, 2017). Put simply: even if the (relative) stimulation intensity is kept constant, the pre-activation of a certain area influences the behavioral outcome and intensities that should otherwise be detrimental to performance may suddenly be facilitatory. Furthermore, there is evidence that TMS early in the task timeline can prime the stimulated area and that even if a brain area is disrupted, other nodes within the network can compensate for it (Hartwigsen, 2018; Klaus & Hartwigsen, 2019). Moreover, disruption of one network node may lead to adaptive plasticity within the network, which may result in up-regulatation to compensate for the perturbation (Brownsett et al., 2014; Geranmayeh et al., 2014, 2017; Nudo, 2003; Turner et al., 2011). Consequently, in our study, regardless of the specific stimulation protocol, TMS may have induced an up-regulation of the whole network or activity shifts to other network nodes that may have overshadowed and compensated for any potential disruptive effect (Hartwigsen, 2018). Such changes are hard to map at the behavioral level and would require additional mapping of neural changes with functional neuroimaging or electroencephalography.

One compelling explanation for the current finding relates to the concept of an optimal level of noise within the neural system. Previous studies have demonstrated that a certain degree of noise can positively affect behavioral performance (Miniussi et al., 2010; Schwarzkopf et al., 2011). The neural noise elicited by NIBS is not completely random and instead influenced by task-related neural activity and the brain's current activation state. The nature of this induced activity can be interpreted as both noise and a critical constituent of the neural signal, contingent upon the specific neuronal circuits that are engaged. When the induced neuronal noise coincides with relevant ongoing neural activity, it may augment the signal, facilitating performance (Ermentrout et al., 2008; Miniussi et al., 2013). Initially proposed as a result of online TMS studies, this concept is generalizable to offline TMS and other NIBS protocols (Abrahamyan et al., 2011; Fertonani & Miniussi, 2017). Offline NIBS, when administered prior to task execution, can transiently activate the targeted neural region, thereby optimizing subsequent task performance (Bergmann & Hartwigsen, 2020). This explanation of a shift towards the TMS-induced optimal signal-to-noise ratio provides a plausible explanation for the observed facilitatory effects in our study. Although not statistically significant, descriptively, the condition-and-perturb condition yielded a larger variance of SSRTs compared with other conditions. If this finding holds true and can be replicated in future work, it may indicate that different network nodes differ in their sensitivity to stimulation.

Furthermore, facilitation can arise when stimulation disrupts processes that compete with task-relevant control operations (typically referred to as “paradoxical facilitation” or “addition-by-subtraction”; Luber & Lisanby, 2014). Following this account, the net effect would be facilitatory and reflect more efficient processing due to a disruption of task-irrelevant processes. Such accounts are complementary to more conservative explanations based on state dependence and network adaptation. Importantly, these interpretations are consistent with the selective shortening of SSRT in the absence of changes in go reaction times, stopping success rates, or trigger failure probabilities.

An additional consideration concerns the temporal specificity of rDLPFC contributions to response inhibition. While the present study targeted reactive stopping by applying online TMS time-locked to stop-signal presentation, the rDLPFC has also been implicated in more anticipatory or context-setting aspects of control, such as maintaining task goals or balancing go and stop demands. To the extent that such processes operate prior to, or independently of, the moment of reactive stopping, they may not be fully captured by online stimulation protocols. Accordingly, rDLPFC stimulation may have influenced stopping performance indirectly by shaping task context or readiness, rather than by directly modulating the inhibitory process itself.

As an alternative explanation, network effects should be considered. Although we targeted specific areas, stimulation-induced disinhibition of distant connected areas has also been proposed as a potential mechanism for cognitive facilitation (Sandrini et al., 2011). Yet, this explanation is less convincing in the context of the present study since the different stimulation protocols did not produce differential effects, and, most importantly, condition-and-perturb TMS did not have an (over)additive effect. Alternatively the "addition-by-subtraction" hypothesis suggests that inhibiting task-irrelevant areas or disrupting distracting stimulus elements can facilitate task-relevant processing (Luber & Lisanby, 2014; Walsh et al., 1998). This explanation seems less likely for our results, given that previous studies have already established the relevance of the targeted areas (Aron et al., 2014; Depue et al., 2016; Lipszyc & Schachar, 2010; Zhang & Li, 2012; Zheng et al., 2008). However, it may explain the tentatively larger variance after condition-and-perturb-TMS.

## Limitations and future work

While TMS facilitated performance in all active stimulation conditions, we have to consider several limitations. First, the stimulation conditions were not fully counterbalanced. We always started with the sham condition because we aimed to establish a baseline first to avoid further drop-out of participants due to low initial performance without TMS. Thus, any comparison of active stimulation conditions compared with sham should be interpreted cautiously, because the comparison of stimulation conditions is always confounded with time. Second, even with a wash-out period of several days in between sessions, we cannot fully rule out a small practice effect in the later sessions that facilitated performance as well. Although a practice effect is in principle a partial alternative explanation of the results, against the background of the literature, this seems unlikely. Practice effects in the SST have been observed mostly after multiple training sessions per week, and even with repeated training, not all studies show a significant practice effect after multiple high-frequency sessions (Berkman et al., 2014; Xu et al., 2020; You et al., 2023). Third, active TMS may have increased the alertness of the participant due to physical stimulation sensations. Nonspecific side-effects, such as intersensory facilitation, would only explain online TMS effects but could not account for the similar condition-and-perturb effects (Duecker et al., 2013; Duecker & Sack, 2013; Terao et al., 1997). Furthermore, because in the online TMS condition, TMS was given concurrently with the stop-signals, it could have caused the participant to wait for the TMS pulse (De Graaf et al., 2009). However, this does not explain the general facilitation across all TMS conditions revealed in the data.

Fourth, one might argue that stimulation-related discomfort could have interfered with task performance and thus contributed to the observed effects. Discomfort ratings differed modestly across conditions—lowest in the sham condition (M = 0.31) and higher in the active stimulation conditions (rIFG: M = 0.97; rDLPFC: M = 0.66; CP: M = 0.90)—but absolute levels were low overall (grand mean ≈ 0.71 on a 0–3 scale). If discomfort or distraction had impaired performance, one would expect slower responses or increased variability in the more uncomfortable conditions (Meteyard & Holmes, 2018). Instead, performance measures were stable across conditions, with the exception of SSRT. Critically, SSRTs were reliably shorter—not longer—in all active stimulation conditions relative to sham. Thus, the conditions associated with greater discomfort were also those showing the largest improvements in stopping latency. Given this opposing direction of effects, stimulation-related discomfort is unlikely to account for the observed SSRT pattern. Nevertheless, the discomfort measure employed was relatively coarse and unspecific; future work using more fine-grained assessments could further evaluate this possibility.

Finally, although TMS is, in general, a NIBS method with high spatial resolution, it is not certain that every participant has received the same stimulation dose in the specific target area. This inter- and even intra-individual variability to TMS may mask potential TMS effects. One way to combat this in future studies would be to combine TMS with a previous fMRI study in which, for each participant separately, the individual peak coordinate can be extracted and the stimulation intensity is adjusted accordingly with electrical field simulations (Numssen et al., 2024). This could lead to more consistent TMS effects for each participant.

## Conclusions

Neither online TMS over the rIFG, rDLPFC, nor the combination of offline and online stimulation produced the expected detrimental effects on stopping performance in the stop-signal task. Instead, all active stimulation conditions were associated with comparable improvements in SSRT relative to sham, with no evidence for differential effects between stimulation targets. Taken together, these results provide no evidence for target-specific or hierarchical causal effects of rIFG and rDLPFC stimulation on reactive stopping performance. Despite a highly powered within-subject design and the use of a condition-and-perturb approach intended to limit compensatory processes, focal perturbation of these prefrontal nodes did not yield selective behavioral impairment.

From a conservative standpoint, this pattern of findings constrains strong causal interpretations of prefrontal TMS effects on response inhibition. While one possible interpretation is that TMS altered the signal-to-noise characteristics of the prefrontal control network in a facilitatory manner, alternative explanations—most notably practice-related effects, order effects, or nonspecific consequences of stimulation, such as arousal or alerting—remain at least partially viable and cannot be conclusively adjudicated on the basis of the present data.

Crucially, however, the absence of differential TMS effects should not be interpreted as a failure of experimental sensitivity. On the contrary, the high statistical power, rigorous control procedures, and fully counterbalanced active stimulation conditions render the present study well suited to place meaningful constraints on focal, target-specific accounts of prefrontal involvement in reactive stopping. Put differently: when compared among themselves, the active stimulation conditions did not modulate performance. Only the comparison of active stimulation to sham resulted in a significant performance difference; however, these comparisons are confounded with the timing of the experimental sessions. Thus, the data does not rule out a general effect of active TMS compared with sham, but any potential effect is not target-specific within the present design. These findings underscore the importance of considering network-level dynamics, learning effects, and nonspecific influences when interpreting behavioral outcomes of prefrontal TMS, and highlight fundamental limits of inferring localized inhibitory mechanisms from perturbation-based approaches in complex control tasks.

Future studies combining concurrent TMS–fMRI, individualized electric-field modeling, and computational approaches may help to disentangle whether subtle, state-dependent, or network-level effects of prefrontal stimulation contribute to inhibitory control, or whether the robustness of stopping behavior reflects compensatory dynamics that limit the causal leverage of focal TMS interventions (for an overview of potential pitfalls in studying cognition using TMS, see Hussain & Freedberg, 2025; Hartwigsen & Silvanto, 2023; Bergmann & Hartwigsen, 2021). Finally, further research integrating systematic protocol comparisons and cumulative evidence will be required to better understand when and how TMS can provide informative causal constraints on models of response inhibition.

## Data Availability

All data can be accessed at OSF via https://osf.io/adnbq/

## Footnotes

1 Power calculations were based on partial eta squared (np^2^). In the *Results* section, generalized eta squared (ges) is reported for ANOVAs to provide a more conservative and comparable estimate of effect size across conditions.

2 Descriptive statistics for resting motor thre and stimulation intensities, including ranges, are provided in the supplementary material; see OSF link at the end of the manuscript.

3 Note that if Condition-Order was added as another factor, the main effect (*p* =.71) and the interaction with stimulation condition (*p* =.19) were not significant. However, order was not completely counterbalanced as Sham was always the first condition.

## Declarations

### Supplemental material

Access at OSF via https://osf.io/adnbq/

### Preregistration

This study was not preregistered.
